# Translation and cross-cultural adaptation of the Pediatric Confusion Assessment Method for the Intensive Care Unit into Brazilian Portuguese for the detection of *delirium* in pediatric intensive care units

**DOI:** 10.5935/0103-507X.20180013

**Published:** 2018

**Authors:** Marizete Elisa Molon, Roberta Esteves Vieira de Castro, Flávia Andrea Krepel Foronda, Maria Clara Magalhães-Barbosa, Jaqueline Rodrigues Robaina, Jefferson Pedro Piva, Pedro Celiny Ramos Garcia, Arnaldo Prata-Barbosa, Elie Cheniaux, Heidi A. B. Smith

**Affiliations:** 1 Faculdade de Medicina, Universidade de Caxias do Sul - Caxias de Sul (RS), Brazil.; 2 Hospital Universitário Pedro Ernesto, Universidade do Estado do Rio de Janeiro - Rio de Janeiro (RJ), Brazil.; 3 Instituto da Criança, Hospital das Clínicas, Faculdade de Medicina, Universidade de São Paulo - São Paulo (SP), Brazil.; 4 Instituto D’Or de Pesquisa e Ensino - Rio de Janeiro (RJ), Brazil.; 5 Faculdade de Medicina, Universidade Federal do Rio Grande do Sul - Porto Alegre (RS), Brazil.; 6 Faculdade de Medicina, Pontifícia Universidade Católica do Rio Grande do Sul - Porto Alegre (RS), Brazil.; 7 Faculdade de Medicina, Universidade Federal do Rio de Janeiro - Rio de Janeiro (RJ), Brazil.; 8 Faculdade de Ciências Médicas, Universidade do Estado do Rio de Janeiro - Rio de Janeiro (RJ), Brazil.; 9 Instituto de Psiquiatria, Universidade Federal do Rio de Janeiro - Rio de Janeiro (RJ), Brazil.; 10 Division of Pediatric Anesthesiology, Vanderbilt University Medical Center - Nashville, TN, United States.

**Keywords:** *Delirium*/diagnosis, Confusion/diagnosis, pCAM-ICU, Intensive care units, pediatric, Translation, Surveys and Questionnaires/standards

## Abstract

**Objective:**

To undertake the translation and cross-cultural adaption into Brazilian Portuguese of the Pediatric Confusion Assessment Method for the Intensive Care Unit for the detection of *delirium* in pediatric intensive care units, including the algorithm and instructions.

**Methods:**

A universalist approach for the translation and cross-cultural adaptation of health measurement instruments was used. A group of pediatric critical care specialists assessed conceptual and item equivalences. Semantic equivalence was evaluated by means of a translation from English to Portuguese by two independent translators; reconciliation into a single version; back-translation by a native English speaker; and consensus among six experts with respect to language and content understanding by means of Likert scale responses and the Content Validity Index. Finally, operational equivalence was assessed by applying a pre-test to 30 patients.

**Results:**

The back-translation was approved by the original authors. The medians of the expert consensus responses varied between good and excellent, except for the feature "acute onset" of the instructions. Items with a low Content Validity Index for the features "acute onset" and "disorganized thinking" were adapted. In the pre-test, the expression "signal with your head" was modified into "nod your head" for better understanding. No further adjustments were necessary, resulting in the final version for Brazilian Portuguese.

**Conclusion:**

The Brazilian version of the Pediatric Confusion Assessment Method for the Intensive Care Unit was generated in agreement with the international recommendations and can be used in Brazil for the diagnosis of *delirium* in critically ill children 5 years of age or above and with no developmental cognitive disabilities.

## INTRODUCTION

*Delirium* is an acute brain dysfunction syndrome caused by a systemic medical condition or brain injury^(1)^ and is among the most frequent complications in intensive care units (ICUs).^(2,3)^ It affects up to 80 % of adults receiving mechanical ventilation (MV) and is associated with prolonged MV and ICU or hospital length of stay and higher mortality, thus leading to increased morbidity and hospital costs.^(4)^ In addition, *delirium* can result in cognitive sequelae and can compromise global long-term functional recovery, even years after hospital discharge.^(5)^ In pediatric ICUs, significant increase in length of hospital stay and the presence of post-traumatic stress symptoms have been associated with *delirium*.^(6)^ In these units, the incidence of *delirium* is estimated at up to 25%, and its prevalence is between 10% and 47%, depending on the population and the characteristics of the unit.^(3,6-8)^ Despite its high prevalence and influence on prognosis of critically ill patients, *delirium* is frequently underdiagnosed.^(9,10)^

Diagnosing *delirium* in critically ill children is specifically difficult due to numerous factors, such as different cognitive stages of development, the effects of acute disease and interventions on the ability to communicate, lack of knowledge and awareness regarding the importance of *delirium*, gaps in education, lack of time for repeated clinical evaluations, scarcity of appropriate instruments, similarity with withdrawal symptoms, and a shortage of available psychiatrists in the ICU. Thus, it is of paramount importance that professionals of these units have access to a valid and reliable tool, that is easily and quickly managed, to assess the primary components of *delirium* in the absence of a psychiatrist.^(11,12)^

Some tools for the diagnosis of *delirium* in the pediatric ICU have been validated and described in the literature, including the Pediatric Anesthesia Emergence *Delirium* (PAED),^(13)^ the Cornell Assessment of Pediatric *Delirium* (CAPD),^(12)^ the Sophia Observation Withdrawal Symptoms-Pediatric *Delirium* Scale (SOS-PD),^(14)^ the Pediatric Confusion Assessment Method for the Intensive Care Unit (pCAM-ICU),^(7)^ and the PreSchool Confusion Assessment Method for the ICU (psCAM-ICU).^(15)^

The pCAM-ICU is an adaptation of the Confusion Assessment Method for the Intensive Care Unit (CAM-ICU),^(16)^ which is used to diagnose *delirium* among adults in ICUs. The pCAM-ICU has been demonstrated to be valid and reliable for administration by non-psychiatrist physicians trained for the diagnosis of *delirium* in children at least 5 years of age, supported or not with MV.^(7)^ Because no tool is yet available for the diagnosis of *delirium* in pediatric ICUs that has been translated and adapted to Portuguese, the objective of the present study was to translate and cross-culturally adapt the pCAM-ICU into the Portuguese language spoken in Brazil, including the algorithm of the tool and a worksheet with instructions for its use.

## METHODS

Before the process was initiated, the developers of the pCAM-ICU^(7)^ from Vanderbilt University Medical Center in Nashville, Tennessee, USA, granted their authorization. The translation and cross-cultural adaptation was undertaken according to the universalist approach of Herdman et al.^(17)^ and Reichenheim and Moraes,^(18)^ who assess six equivalences: conceptual, items, semantic, operational, measurement, and functional. These steps are similar to those recommended by the Principles of Good Practice for the Translation and Cultural Adaptation Process for Patient-Reported Outcomes of the International Society for Pharmacoeconomics and Outcomes Research (ISPOR),^(19)^ though with diverging terminology. The present study focused on conceptual, item, semantic, and operational equivalence and did not consider measurement and functional equivalence. The latter is underway and will be part of the research on validity and reliability of the pCAM-ICU.

Pediatric critical care specialists performed evaluations of conceptual and item-equivalence, which involved a review of the literature and group meetings. For the evaluation of semantic equivalence, two independent translations of the pCAM-ICU tool from English to Brazilian Portuguese were performed by two pediatric critical care intensivists who were fluent in English. These translations were merged into a preliminary version, which was then back-translated into English by a North American translator fluent in Brazilian Portuguese. The original authors of the tool revised and approved the back-translated English version. These translations included the algorithm of the pCAM-ICU tool and the worksheet containing instructions for its administration.

To complement the semantic evaluation, the reviewed preliminary version of the tool and the instructions for its administration were submitted to independent appraisal by six pediatric critical care experts fluent in English. These experts received by email the original pCAM-ICU, the preliminary Portuguese version, and a questionnaire addressing language and content understanding, beginning at the first instruction of the 2^nd^ step, and including each of the four features of the translated tool that comprise the pCAM-ICU *per se* because the 1^st^ step refers to the application of the Sedation Scale and the Richmond Sedation-Agitation Scale (RASS). The questionnaire contained open questions with the possibility to suggest changes, along with Likert scale responses with six possible answers (1 - very poor, 2 - poor, 3 - fair, 4 - good, 5 - very good, and 6 - excellent). The answers and suggested changes were discussed and compiled in three consensus meetings and gave rise to a new version.

The level of consensus among the experts was estimated with the median of the Likert responses regarding understanding of the 2^nd^ step and of each of the four features, and with the Content Validity Index (CVI) for the adaptation of measuring instruments, which was calculated for the same items.^(20)^ The CVI, which also uses a Likert scale, assesses the percentage of agreement among the evaluators regarding specific aspects of the instrument and their items so that each item can be examined separately, as can the instrument as a whole. The CVI is calculated by adding the items "good, very good and excellent", divided by the total number of raters. The suggested minimum agreement is 0.8.^(20)^

Prior to the evaluation of operational equivalence, researchers received proper training by watching videos in English from the *delirium* study group from Vanderbilt University Medical Center and performed simulations on other researchers with the Brazilian version of the pCAM-ICU in the Portuguese language. A pre-test was then performed by administering this new version to 30 inpatients from either of the participating pediatric ICUs: *Hospital Quinta D'Or* and *Hospital Caxias D'Or* (*Rede D'Or São Luiz/Instituto D'Or de Pesquisa e Ensino* - IDOR). Participants were aged between 5 and 18 completed years. Their legal guardians signed an informed consent form or, if possible, the participants themselves signed the terms of informed assent. Considering that the tool requires patient participation, those with chronic encephalopathy or neuropsychological developmental delays and those who were not able to understand Portuguese were excluded. *Stata* 11 software (*Stata Corp LP*^®)^ was used for data entry and analysis.

The present study has been approved by the research Ethics Committee of the *Hospital Universitário Pedro Ernesto* (HUPE) of *Universidade do Estado do Rio de Janeiro* (UERJ) and the IDOR, under the report numbers 1.726.576 (CAAE 34302114.7.1001.5259) and 1.736.635 (CAAE 34302114.7.3001.5249), respectively.

### Description of the Pediatric Confusion Assessment Method for the Intensive Care Unit

**Figure 1:**
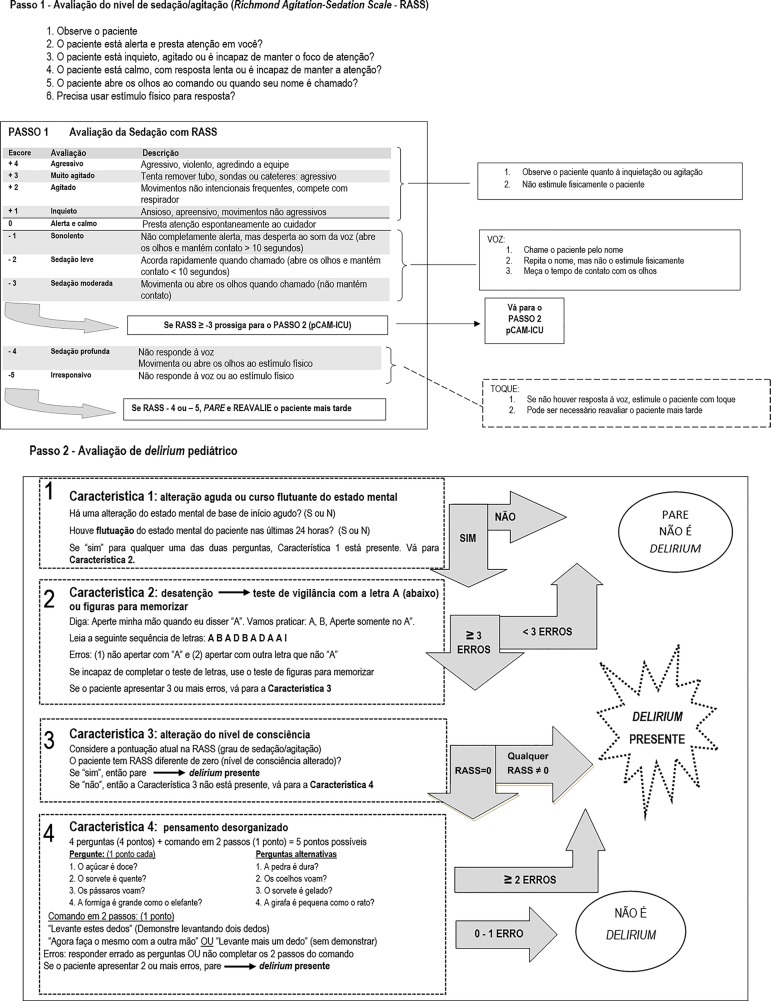
Pediatric Confusion Assessment Method for the Intensive Care Unit. RASS - Richmond Agitation-Sedation Scale; pCAM-ICU - Pediatric Confusion Assessment Method for the Intensive Care Unit; Y - yes; N - no. Adapted from: Smith HA, Boyd J, Fuchs DC, Melvin K, Berry P, Shintani A, et al. Diagnosing delirium in critically ill children: Validity and reliability of the Pediatric Confusion Assessment Method for the Intensive Care Unit. Crit Care Med. 2011;39(1):150-7.^(7)^

**Figure 2:**
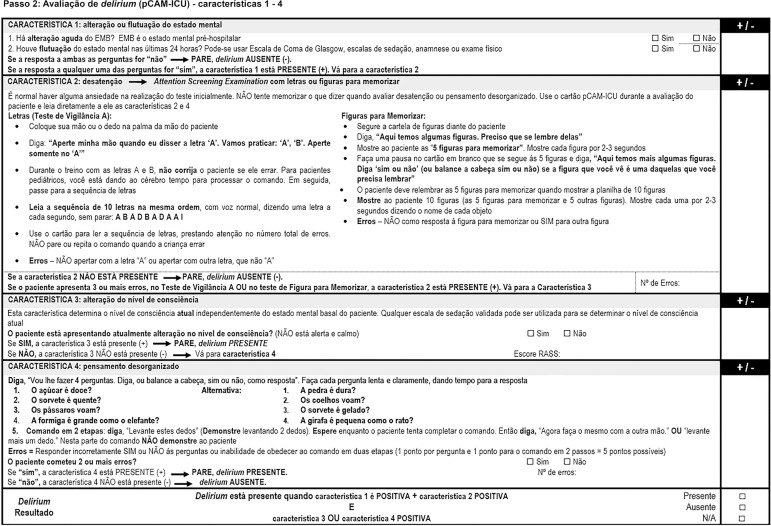
Worksheet for the administration of the Pediatric Confusion Assessment Method for the Intensive Care Unit. pCAM-ICU - Pediatric Confusion Assessment Method for the Intensive Care Unit; RASS - Richmond Agitation-Sedation Scale.

The administration of the pCAM-ICU (Figures 1 and 2) requires an adequate level of consciousness to trigger answers. Thus, the 1^st^ step in the assessment of *delirium* is to determine the level of consciousness by means of the RASS scale, which is divided into four levels of anxiety or agitation (+1 to +4), one level that indicates a state of alertness and calmness (zero), and five levels of sedation (-1 to -5). Level -4 indicates patients who do not respond to verbal stimulation but who open and move their eyes upon physical stimulation. Level -5 indicates no response to verbal or physical stimulation. Thus, participants with RASS scores of -4 or -5 cannot be submitted to the evaluation of *delirium*. If their RASS score is above -4 (-3 to +4), evaluators proceed to the 2^nd^ step and administer the pCAM-ICU. For a positive diagnosis of *delirium*, the patient must exhibit features 1 and 2 (acute onset and inattention) and either 3 (altered level of consciousness) or 4 (disorganized thinking). Feature 1 is positive if any changes or variations in the mental status baseline (MSB) within a period of 24 hours are present. Feature 2 is assessed by means of the Attention Screening Examination (ASE), either using the letters test or, if this is not possible, using the pictures test. The latter is applied with the original set of picture cards, which can be downloaded from the internet.^(21)^ Because these tests do not require verbal responses, they are ideal for patients on MV. If the score is below 8 (maximum = 10), inattention is present. Any level of consciousness other than "alert and calm" indicates a positive feature 3. If this feature is negative (RASS = 0), feature 4 is assessed (disorganized thinking), which is composed of four questions (two sets of four questions, alternating between sets for each assessment of the same patient) and a 2-step command, totaling five points (the accomplishment of both commands correspond to 1 point). Feature 4 is positive when the score is below or equal to 3, *i*.*e*., the patient fails at least two questions, or one question and the 2-step command.^(9,12)^

## RESULTS

### Semantic equivalence

The back-translation of the translated Portuguese version into English was approved by the original authors of the pCAM-ICU. Regarding expert consensus, most items exhibited answers between "good" and "excellent" (4 to 6) for both the algorithm and the pCAM-ICU instructions worksheet (Table 1). In the initial translated version the definition of "MSB" in the feature 1 of the instructions worksheet (Figure 2) had been excluded, which created doubts among the experts during the consensus meetings. Thus, and in agreement with the original English version, we added the definition of MSB as "pre-hospital mental status" and specified the methods to assess the mental status in the past 24 hours, *i*.*e*., the Glasgow Coma Scale (GCS), sedation scales, anamnesis, or physical exam. Discrepant results with CVIs < 0.8 in the questions of feature 2 and the commands of feature 4 were reviewed and corrected according to suggestions from the experts, who then approved the version that was used in the pre-test. We rearranged both sets of four questions so that they became more similar to the original algorithm in English and so that the alternation between the two sets became clearer, and we performed discrete changes in phrasing of both commands to render them clearer to patients.

**Table 1:** Results of expert consensus for the evaluation of the first translated version of the Confusion Assessment Method for the Intensive Care Unit into Brazilian Portuguese

	pCAM-ICU algorithm		Instructions for use	
	Median (IQR 25 - 75%)	Content Validity Index (%)	Median (IQR 25 - 75%)	Content Validity Index (%)
2^nd^ step	4.5 (4 - 5)	0.83	-	-
Feature 1	5.5 (4 - 6)	1	2 (2 - 3)	0.17
Feature 2				
Letters test	4 (4 - 5)	0.83	4 (3 - 4)	0.67
Pictures test	5 (4 - 5)	0.83	4 (3 - 5)	0.67
Feature 3	4.5 (4 - 6)	1	4.5 (4 - 6)	1
Feature 4				
Questions	3.5 (3 - 5)	0.5	4.5 (4 - 5)	0.83
2-step command	3.5 (3 - 4)	0.5	4.5 (4 - 5)	0.83

pCAM-ICU - Pediatric Confusion Assessment Method for the Intensive Care Unit; IQR - interquartile range. Median with respective interquartile range and Content Validity Index.

### Operational equivalence

The characteristics of patients who participated in the pre-test are listed in table 2. Each application took less than 1 minute. Three patients were drowsy, which hampered tool administration. One patient presented with amaurosis and, thus, was not submitted to the ASE pictures test. One child had difficulties with understanding the expression "signal with your head", which was better interpreted after being changed to "nod your head". After this change, all children reported that they understood all questions and commands (100%).

**Table 2:** Pre-test clinical patient characteristics (n = 30)

Clinical characteristics	
Age*	8 (6 - 12)
Sex	
Female	15 (50.0)
Male	15 (50.0)
Age range	
Pre-school (5 years)	7 (23.3)
Elementary (6 - 9 years)	10 (33.3)
Adolescents (≥ 10 years)	13 (43.3)
Type of hospital admission	
Surgery	3 (10.0)
Clinical	26 (86.7)
Neurosurgery	1 (3.3)
Diagnoses	
Metabolic	2 (6.7)
Neurological	2 (6.7)
Onco-hematological	5 (16.7)
Respiratory	17 (56.7)
Other	4 (13.3)
Sepsis	2 (6.7)
RASS*	0 (0-0)
Length of stay in the pediatric ICU (days)*	2 (1 - 3)
Ventilatory support at the time of evaluation	
Ambient air	17 (56.7)
Nasal cannula	6 (20.0)
Hudson mask	2 (6.7)
Continuous NIV	2 (6.7)
Intermittent NIV	3 (10.0)
Use of vasoactive amines at the time of evaluation	2 (6.7)
Use of sedo-analgesics at the time of evaluation	0 (0)
Previous use of midazolam/fentanyl under continuous infusions	1 (3.3)
Duration (days)	6/8
Maximum dose (midazolam mg/kg/h / fentanyl mcg/kg/h)	0.4/2.0
Time of suspension (hours)^†^	48/4
PRISM*	0.7 (0.4 - 1.2)
PIM-2*	1.2 (0.4 - 1.7)
Diagnosis of *delirium*	0 (0)

RASS - Richmond Agitation-Sedation Scale; ICU - intensive care unit; NIV - noninvasive ventilation; PRISM - Pediatric Risk of Mortality; PIM-2 - Pediatric Index of Mortality-2.

* Median (IQ25-75);

† time spent between suspension of the drug and evaluation of *delirium*. Results are expressed as n (%) or medians (interquartile ranges).

The final Portuguese version of the pCAM-ICU, which contemplated the results of conceptual, item, semantic, and operational equivalence, is shown in figures 1 and 2.

## DISCUSSION

This is the first Brazilian study to translate and cross-culturally adapt a tool for the diagnosis of *delirium* in pediatric ICUs, maintaining agreement with international guidelines to ensure the quality of results. The final version of the translated and adapted pCAM-ICU into Brazilian Portuguese exhibited evidence of good levels of acceptance and verbal understanding.

The lack of diagnostic tools hampers the early identification of any given disease. Undertaking translation and cross-cultural adaption of existing tools, rather than elaborating new tools, is a way of addressing this demand, allowing for levels of reliability and validity like those of the original tool. Furthermore, adaptations can be used as a reference in research involving several countries for a better understanding of the disease in different cultures and languages.^(22)^

The use of a diagnostic tool created in different cultural settings must be preceded by thorough evaluations between the original and translated versions of the instrument. This involves not only different countries and languages but also different regions of the same country. In Brazil, for example, the diverse colloquial expressions adopted in one region might not be accepted in another. Linguistic changes can occur in one and the same population over the years, thus requiring periodic adaptations. Literal translations, without adequate operationalization, can compromise the information, which hampers comparative studies.^(18)^ During the process of the present study, we ensured that these recommendations were met, both for the pCAM-ICU tool and for the instructions of administration.

Some authors have affirmed the relevance of a minimum of two translators whose vernacular language is the target language of the translation (in this case, Brazilians), and who perform the translation independently, to avoid any excess of a single writing style. They must be aware of the purpose of the tool so that the meanings of terms are in agreement with the context.^(17,18,22)^ The profiles of both translators in the present work matched these descriptions. In contrast, the back-translator received little information about the tool to avoid any bias in the correction of the translation.^(17,22)^

Expert consensus was important to finalize the semantic evaluation and resulted in some adjustments to the tool that was to be administered in the pre-test. Operational equivalence was assessed in a pre-test with 30 patients, as recommended by Guillemin et al.,^(23)^ who suggested that this equivalence is assessed on 15 to 30 patients of the target population. Both instrument acceptance and understanding were good among participating patients.

The pCAM-ICU is a modification of the CAM-ICU to detect *delirium* among children 5 years of age or above. It includes color pictures and questions with good discriminative capacity, such as "Is ice cream hot?". The original tool exhibited high sensitivity (83 %), specificity (99 %), and reliability (κ = 0.96).^(7)^ This tool has already been translated into German and Spanish^(24,25)^ and was chosen for our study because it is the adaptation of a globally accepted tool used for adult ICUs^(16)^ and because it is the first tool to be validated for the diagnosis of *delirium* in pediatric ICUs, in its three modalities, according to psychomotor activity (hypoactive, hyperactive, and mixed).^(7)^ Considering the difficulties in recognizing *delirium* based only on non-instrumented clinical evaluations, a validated diagnostic tool would enable non-psychiatric physicians to recognize most of the cases, especially the hypoactive form.^(5)^

One limitation of the present work was the absence of patients on invasive MV in the pediatric ICUs when the pre-test was conducted, thus hampering the evaluation of difficulties specific to this patient population regarding tool administration. However, pediatric patients on invasive MV are usually sedated to a certain degree, which precludes the administration of the pCAM-ICU. Moreover many of the tested patients exhibited other types of ventilatory support frequently used in pediatric ICUs. A further limitation of this study is the fact that the tool was back-translated by a single back-translator, as ideally, this step should be performed by the same number of people as there were translators.^(23)^ Still, semantic equivalence was assessed by several experts, thus reducing the possibility of bias.

The recent development of tools to diagnose *delirium* is a promising step for its detection in pediatric ICUs and enables the identification of modifiable risk factors and of short- and long-term effects of *delirium* in these patients. The current guidelines of the American College of Critical Care Medicine and of the Society of Critical Care Medicine (2013) recommend routine monitoring of *delirium*, at least once per nursing shift.^(26)^ These guidelines were revisited by the European Society of Pediatric and Neonatal Intensive Care (ESPNIC) in 2016, which then recommended frequent and specific screening in pediatric ICUs by means of a validated tool.^(27)^

## CONCLUSION

The translation and cross-cultural adaptation of the pCAM-ICU tool into Brazilian Portuguese was performed in agreement with international norms and originated the Brazilian version, which will allow for the continuity of validation and reliability studies on children with at least 5 years of cognitive and chronological age and for its application in research on this group of inpatients in pediatric intensive care units, thus contributing to the diagnosis and prevention of *delirium* in the country.
